# Scoping Review of Culinary Interventions in Schools: Mapping Practices to Promote Food Education and Equity

**DOI:** 10.1111/josh.70243

**Published:** 2026-10-15

**Authors:** Bruno Bastos, Luis Phillipe Nagem Lopes, Joice Soltosky Cunha, Letícia Ferreira Tavares

**Affiliations:** ^1^ Department of Gastronomy Josué de Castro Nutrition Institute, Federal University of Rio de Janeiro Rio de Janeiro Brazil; ^2^ Pharmacy School Federal University of Rio de Janeiro Rio de Janeiro Brazil; ^3^ Institute of Social Medicine State University of Rio de Janeiro Rio de Janeiro Brazil; ^4^ Postgraduate Program in Pharmaceutical Sciences University of Sorocaba São Paulo Brazil; ^5^ State University of Rio de Janeiro Rio de Janeiro Brazil

**Keywords:** children, culinary, education, school, scoping review

## Abstract

**Background:**

School‐based culinary interventions promote healthy eating, but evidence on their implementation and educational contributions remains fragmented. This study mapped culinary practice interventions as food education strategies for children and adolescents in schools.

**Methods:**

Following Joanna Briggs Institute guidelines, we conducted a scoping review of studies involving school‐aged participants in culinary interventions. Searches were conducted in October 2024 across PubMed, ERIC, Virtual Health Library, Medline, Scopus, and grey literature without date restrictions. Study selection, data extraction, and intervention classification were performed independently by two reviewers.

**Findings:**

Fifty‐five studies met the inclusion criteria. All were conducted in high‐income countries and were predominantly quantitative (*n* = 43; 78.2%). Interventions mainly targeted elementary students (*n* = 47; 85.5%), were often integrated into curricula (*n* = 26; 47.3%), and emphasized attitudinal and psychosocial outcomes (*n* = 48; 87.3%). Teachers were the main facilitators (*n* = 45; 81.8%), while only three studies (5.5%) involved multidisciplinary teams including Education, Gastronomy, and Nutrition professionals.

**Implications for School Health Policy, Practice, and Equity:**

Future interventions should address broader equity factors, engage multidisciplinary teams, and broaden food education.

**Conclusions:**

School‐based culinary interventions support comprehensive food and nutrition education, but greater attention to equity and multidisciplinary collaboration is needed.

## 1 Introduction

Childhood and adolescence are critical periods for developing eating habits that often persist into adulthood [1, 2, 3]. Food choices during these stages are shaped by family practices, food culture, and exposure to the marketing of ultra‐processed foods [4, 5]. Consequently, school‐based food education has become an important strategy to promote healthy eating habits and food literacy among children and adolescents [4, 6, 7].

Schools are strategic settings for health promotion because they reach students, teachers, staff, and families while supporting intellectual and civic development [8]. Within this context, school‐based culinary interventions have expanded considerably in recent years, frequently incorporating cooking activities alongside school gardening and physical education [9, 10, 11]. These interventions include food preparation, cooking, food tasting, and healthy eating education, delivered by teachers, food service staff, or other school personnel in curricular or extracurricular settings [9]. However, they remain highly heterogeneous regarding educational objectives, pedagogical approaches, activity frequency, use of cooking facilities, and the professionals involved [10]. Understanding this diversity is essential to characterize how culinary interventions are implemented and how they contribute to food education [11].

Despite their growing adoption, integrating culinary activities sustainably into school curricula and students's daily routines remains challenging [12]. Furthermore, many initiatives continue to emphasize the biological and nutritional dimensions of food, with less attention to its social, cultural, economic, environmental, and political dimensions [13]. Food is increasingly recognized as a socioscientific issue that encompasses nutrition, sustainability, food security, culture, inequality, and social justice [14, 15]. Culinary education offers opportunities to connect these dimensions with everyday practices, fostering critical reflection while linking food systems, health, society, and the environment [16, 17]. Gastronomy professionals may further strengthen these educational processes by contributing culinary expertise and practical learning experiences.

Previous reviews have primarily focused on the effects of school‐based food education on eating behaviors and dietary habits [18, 19, 20], while some have also examined teacher training, the role of nutritionists, and experiences involving low‐income families [19, 21]. However, no review has comprehensively mapped how school‐based culinary interventions are designed and implemented across educational contexts, including their didactic objectives, culinary competencies, involvement of gastronomy professionals, integration with curricular subjects [22], and equity considerations, such as the representation of ethnic‐racial and gender minorities [23]. Moreover, the relationship between culinary interventions and core educational subjects has received limited attention [22]. To address this knowledge gap, this scoping review aims to map school‐based culinary practice interventions used as food education strategies for children and adolescents.

## 2 Methods

### 2.1 Study Design

This scoping review followed the Preferred Reporting Items for Systematic Reviews and Meta‐Analyses extension for Scoping Reviews (PRISMA‐ScR) (Table S1) [24]. A scoping review was selected because this methodology is recommended for mapping a specific topic according to the volume, nature, and key characteristics of primary research [25]. We followed the Joanna Briggs Institute (JBI) methodological guidelines [26]. JBI is an international organization that develops globally recognized methodologies for evidence synthesis and evidence‐informed decision‐making [26]. The review protocol was registered with the Open Science Framework (OSF) in May 2025, before data extraction and analysis [27]. To enhance disciplinary relevance, a nutrition professional (LFT) and a gastronomy professional (BB) contributed to all stages of the review, including protocol development, eligibility criteria, data extraction, and interpretation of findings.

### 2.2 Eligibility Criteria

The research question was guided by the Population, Concept, and Context (PCC) framework [26]:

#### 2.2.1 Population

Children and adolescents were defined as individuals aged 0–19 years. Studies including adults were also considered when interventions targeted children and adolescents or when outcomes for children and adults were reported separately. No restrictions were applied to primary study design; all designs reporting original empirical data were eligible.

#### 2.2.2 Concept

Culinary practice interventions, defined as group activities that promote learning about food in a dynamic and interactive manner [28]. These activities should provide a space for the development of cooking skills, the preservation of food culture, and/or the promotion of autonomy in food choices [29]. Studies involving only tasting, games, or playful activities without culinary practice were excluded.

#### 2.2.3 Context

Interventions conducted in educational settings. The setting classification followed the terminology adopted by the original studies and included schools, preschools, kindergartens, and other early childhood education environments. Multi‐context studies were also considered when culinary activities occurred within an educational institution.

### 2.3 Exclusion Criteria

Studies were excluded if they: (1) did not involve children or adolescents as the target population; (2) were conducted exclusively outside school settings; (3) described food‐related activities without a culinary practice component, such as food tasting, games, or other playful activities not involving food preparation; (4) did not report original empirical data, including reviews, editorials, commentaries, opinion papers, protocols, and conference abstracts. For studies including both children/adolescents and adults, only those in which interventions targeted children and adolescents or outcomes were reported separately were eligible.

### 2.4 Information Sources

The search strategy was developed and validated by a librarian and implemented in October 2024 (Table S2) [27]. Databases searched included Medline (via PubMed), ERIC, Scopus, and the Virtual Health Library. Grey literature was searched via Google Scholar in private mode, and the first 200 non‐peer‐reviewed records, including theses, dissertations, and other relevant documents, were screened for eligibility to reduce algorithmic bias [30].

### 2.5 Study Selection and Data Extraction

Study selection was conducted in two stages: title and abstract screening, followed by full‐text review, using Rayyan. Rayyan is a web‐based application that supports study screening and selection in evidence synthesis by facilitating collaboration among reviewers and management of inclusion and exclusion decisions.

Two reviewers (BB and LPNL) independently screened all titles, abstracts, and full‐text records following a calibration exercise designed to ensure consistency in the application of the eligibility criteria. Disagreements were resolved by consensus or, when necessary, by consultation with a third reviewer (LFT) [26].

Data extraction was performed independently by two reviewers (BB and LPNL) using Microsoft Excel 2016. Information was extracted on the study (e.g., country, year of publication, study design, and participant characteristics), culinary practice interventions (e.g., duration, frequency, facilitators, setting, and educational components), and how food was addressed (e.g., nutritional, cultural, environmental, and social dimensions). Details of the extracted variables are provided in the Supporting Information (Table S3).

### 2.6 Categorization of Findings

Equity‐related information on participant recruitment and study populations was extracted and categorized using the PROGRESS‐Plus framework (Place of residence, Race/ethnicity, Occupation, Gender/sex, Religion, Education, Socioeconomic status, Social capital, and additional factors associated with potential health inequities [Plus]), a widely used framework in health research for identifying social and contextual factors associated with health inequities [31].

Studies were also classified according to the income level of the country in which the study was conducted, based on the World Bank income classification [32]. Countries were categorized as low‐, lower‐middle‐, upper‐middle‐, or high‐income economies according to their Gross National Income per capita.

The outcomes reported by the included studies and assessed in relation to culinary practice interventions were categorized into four dimensions: conceptual, procedural, attitudinal, and anthropometric. Conceptual outcomes relate to theoretical knowledge, explanations, and foundations students are expected to understand (e.g., nutrition knowledge and information about food origins) [16]. Procedural outcomes involve cognitive and motor actions aimed at achieving objectives through specific methods and techniques, such as culinary skills and communication about healthy eating with peers [16]. Attitudinal outcomes refer to subjects' tendencies and predispositions guided by norms and values, including positioning, attitudes, and actions emerging from concrete situations and contexts, fostering self‐reflection (e.g., food preferences, willingness to try new foods, enjoyment and enthusiasm in cooking) [16]. Anthropometric outcomes involve systematic measurement of body dimensions and metabolic assessments, including obesity indices, body composition, physical fitness, and metabolic levels [33].

Interventions were also categorized according to the dimensions of food addressed: sociocultural, socio‐environmental, and psychosocial [34]. Sociocultural dimensions included activities mobilizing societal meanings, practices, and symbols associated with food (e.g., cultural belonging, traditions, social values). Socio‐environmental dimensions involve environmental, territorial, and ecological conditions related to food (sustainability, climate change, cultivation methods). Psychosocial dimensions addressed behavioral, subjective, emotional, and relational aspects of food (e.g., social interactions, habit changes, interest in eating).

Culinary skills were categorized into: (1) food handling and preparation techniques; (2) hygiene and food safety practices; (3) label reading, recipes, dietary guidelines, and food information; and (4) sensory and physicochemical food analysis [35]. All categorizations were conducted by one reviewer and verified by another.

### 2.7 Data Synthesis

Data were summarized using absolute and relative frequencies, following guidance for systematic reviews without meta‐analysis [36]. Data were managed in Microsoft Excel 2016.

## 3 Findings

### 3.1 Study Selection

As shown in Figure 1, the search identified 3435 records, of which 55 studies were included. Excluded studies classified as wrong population (*n* = 8) did not focus on children or adolescents; wrong concept (*n* = 52) did not involve culinary practice interventions; and wrong context (*n* = 24) were not conducted in educational settings. Studies classified as unavailable data (*n* = 9) were conference abstracts with insufficient data for inclusion (Table S4).

**FIGURE 1:**
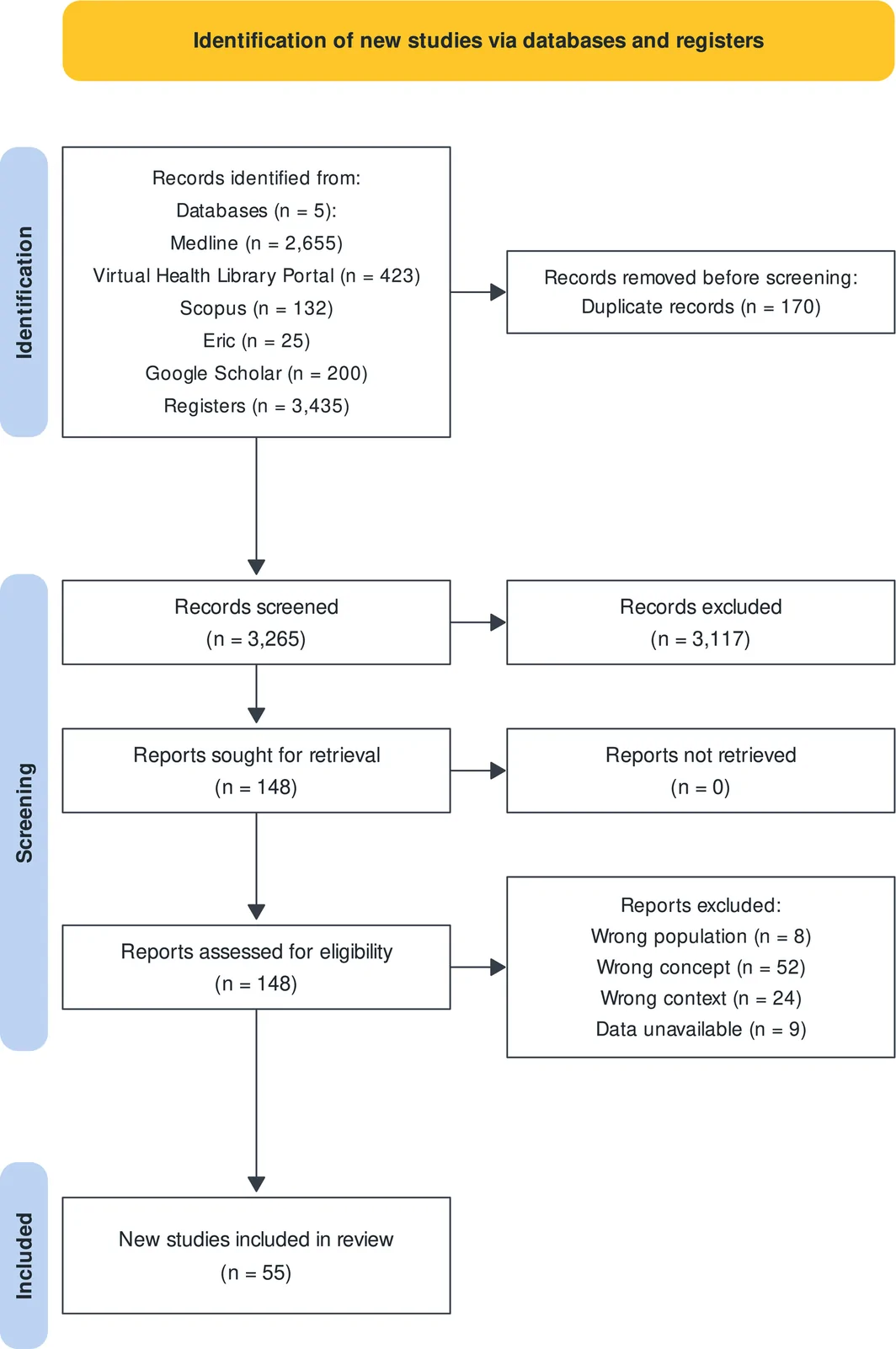
Selection process of studies on culinary interventions for children and adolescents in school environments.

### 3.2 Study and Participant Characteristics

The 55 included studies, published between 1998 and 2024, are summarized in Table 1. Most used quantitative designs (*n* = 43; 78.2%), mainly quasi‐experimental (*n* = 26; 47.3%) or randomized controlled designs (*n* = 14; 25.5%). Participants were mostly elementary school students (*n* = 47; 85.5%), aged 3–19 years. Sample sizes ranged from 6 to 5336, and 27 studies (49.1%) had predominantly female participants.

**TABLE 1:** Characteristics of the included studies addressing culinary practice interventions for children and adolescents in the school setting (*n* = 55 studies).

References	Study design	Number of participants (% female)	Education level	Equity (PROGRESS‐Plus)	Country	Country income level	Outcome categorization (anthropometric attitudinal, conceptual and procedural)
Liquori et al. 1998 [37]	Quasi‐experimental (before and after)	590 (NR)	Elementary education	Income: low income	United States	High income	Attitudinal, conceptual and procedural
Yoshida et al. 2008 [38]	Longitudinal (prospective)	154 (18.1)	Elementary education	NR	Japan	High income	Anthropometric and attitudinal
Walters and Stacey, 2009 [39]	Methodological study	4400 (NR)	Elementary education	Ethnicity: Hispanic Income: low income	United States	High income	Procedural
Lukas and Cunningham‐Sabo, 2011 [40]	Focus groups	178 (48.3)	Elementary education	Income: low income Ethnicity: Hispanic	United States	High income	Procedural
Block et al. 2012 [41]	Quasi‐experimental (before and after)	592 (NR)	Elementary education	Income: low income	Australia	High income	Attitudinal and procedural
Beaulieu and Godin, 2012 [42]	Theory‐based study	241 (NR)	High school education	NR	Canada	High income	Attitudinal
Caraher et al. 2013 [43]	Quasi‐experimental (before and after)	169 (53.2)	Elementary education	NR	England	High income	Attitudinal and procedural
Gibbs et al. 2013 [44]	Quasi‐experimental (before and after)	743 (53.1)	Elementary education	Location: rural	Australia	High income	Attitudinal, conceptual and procedural
Cunningham‐Sabo and Lohse, 2013 [45]	Experimental (clinical trial)	257 (54)	Elementary education	Ethnicity: Hispanic Income: low income	United States	High income	Attitudinal and procedural
Gibbs et al. 2013 [46]	Quasi‐experimental (before and after)	592 (NR)	Elementary education	Location: rural	Australia	High income	Attitudinal and procedural
Alexander et al. 2014 [47]	Longitudinal (prospective)	561 (NR)	Elementary education	Income: low income	United States	High income	Anthropometric
Cunningham‐Sabo and Lohse, 2014 [48]	Quasi‐experimental (before and after)	1230 (50.4)	Elementary education	Ethnicity: Hispanic Income: low income	United States	High income	Attitudinal and procedural
Roura et al. 2014 [49]	Quasi‐experimental (before and after)	2516 (NR)	High school education	NR	Spain	High income	Attitudinal and conceptual
Carraway‐Stage et al. 2015 [50]	Quasi‐experimental (before and after)	473 (53.2)	Elementary education	Location: rural	United States	High income	Conceptual
Martinez et al. 2015 [11]	Experimental (clinical trial)	304 (51.6)	Elementary education	Income: low income	United States	High income	Anthropometric, attitudinal, and procedural
Utter et al. 2015 [51]	Focus groups	6 (NR)	High school education	Other: conducted in alternative education schools, which serve students who had to leave mainstream schooling.	New Zealand	High income	Anthropometric and attitudinal
Ritchie et al. 2015 [52]	Quasi‐experimental (before and after)	118 (NR)	Elementary education	NR	Australia	High income	Attitudinal, conceptual and procedural
Ensaff et al. 2015 [53]	Focus groups	76 (61.8)	Elementary education	NR	England	High income	Attitudinal and conceptual
Davis et al. 2016 [54]	Experimental (clinical trial)	304 (51.6)	Elementary education	Income: low income	United States	High income	Anthropometric and attitudinal
Jarpe‐Ratner et al. 2016 [55]	Quasi‐experimental (before and after)	271 (65)	Elementary education	Income: low income	United States	High income	Attitudinal, conceptual and procedural
D'Adamo et al. 2016 [56]	Longitudinal (prospective)	110 (64.5)	High school education	Ethnicity: predominantly African American	United States	High income	Attitudinal and procedural
Battjes‐Fries et al. 2016 [57]	Quasi‐experimental (before and after)	800 (51.7)	Elementary education	NR	Netherlands	High income	Attitudinal, conceptual and procedural
Ensaff et al. 2017 [58]	Longitudinal (prospective)	338 (NR)	Elementary education	NR	United Kingdom	High income	Attitudinal and procedural
Price et al. 2017 [59]	Cross‐sectional	531 (NR)	High school education	Income: low income	United States	High income	Attitudinal and procedural
Zahr and Sibeko, 2017 [60]	Quasi‐experimental (before and after)	100 (48)	Elementary education	NR	Canada	High income	Attitudinal and procedural
Segrott et al. 2017 [61]	Case study	1342 (NR)	Elementary education	Income: low income	England	High income	Attitudinal and procedural
Wolfe et al. 2018 [62]	Quasi‐experimental (before and after)	5336 (51)	Elementary education	Income: low income	United States	High income	Attitudinal
White et al. 2018 [63]	Focus groups	8 (62.5)	Elementary education	Income: low income Gender	United States	High income	Conceptual
Adab et al. 2018 [64]	Experimental (clinical trial)	1397 (48.9)	Elementary education	NR	England	High income	Anthropometric and attitudinal
Bai et al. 2018 [65]	Quasi‐experimental (before and after)	71 (49.2)	Elementary education	NR	South Korea	High income	Attitudinal and procedural
Landry et al. 2019 [10]	Experimental (clinical trial)	290 (51)	Elementary education	Income: low income	United States	High income	Anthropometric, attitudinal and procedural
Azzopardi, 2019 [66]	Experience report	NR	Outro	Others: diagnosis of mental and neurodevelopmental disorders	England	High income	Procedural
Davis et al. 2019 [67]	Experimental (clinical trial)	3132 (47.4)	Elementary education	Ethnicity: Black and Hispanic	United States	High income	Anthropometric and attitudinal
Højer et al. 2020 [68]	Participant observation	132 (59.1)	Elementary education	NR	Denmark	High income	Attitudinal and procedural
Ali et al. 2020 [69]	Quasi‐experimental (before and after)	40 (52.5)	Elementary education	Income: low income	Spain	High income	Anthropometric, attitudinal, conceptual and procedural
Kim and Park, 2020 [70]	Quasi‐experimental (before and after)	202 (51)	Elementary education	NR	South Korea	High income	Attitudinal
Takacs et al. 2020 [71]	Quasi‐experimental (before and after)	229 (55.5)	Elementary education	NR	Hungary	High income	Anthropometric, atitudinal, conceptual and procedural
Davis et al. 2021 [72]	Experimental (clinical trial)	2721 (52.8)	Elementary education	Ethnicity: Hispanic	United States	High income	Anthropometric and attitudinal
Maiz et al. 2021 [73]	Quasi‐experimental (before and after)	202 (43)	Elementary education	NR	Spain	High income	Anthropometric, attitudinal, conceptual and procedural
Landry et al. 2021 [74]	Experimental (clinical trial)	738 (52)	Elementary education	Ethnicity: Black and Hispanic	United States	High income	Attitudinal
Yoshii et al. 2021 [75]	Quasi‐experimental (before and after)	250 (44.8)	Elementary education	NR	Japan	High income	Attitudinal and procedural
Ng et al. 2022 [76]	Experimental (clinical trial)	83 (61.4)	Elementary education	NR	Malaysia	Upper middle income	Attitudinal, conceptual and procedural
LeBlanc et al. 2022 [77]	Quasi‐experimental (before and after)	326 (53.3)	High school education	Location: rural	Canada	High income	Attitudinal and procedural
Elsborg et al. 2022 [78]	Quasi‐experimental (before and after)	640 (48)	Elementary education	Income: low income Localização: rural	Denmark	High income	Attitudinal e conceptual
Davis et al. 2023 [9]	Experimental (clinical trial)	695 (55.82)	Elementary education	Ethnicity: Black and Hispanic	United States	High income	Anthropometric
Davis et al. 2023 [79]	Experimental (clinical trial)	3114 (48.6)	Elementary education	Ethnicity: Black and Hispanic	United States	High income	Attitudinal
Pini et al. 2023 [80]	Focus groups and interviews	10 (60)	Elementary education	Income: low income	United Kingdom	High income	Attitudinal and procedural
Jeans et al. 2023 [81]	Experimental (clinical trial)	451 (47)	Elementary education	Ethnicity: Black and Hispanic	United States	High income	Attitudinal
Cunningham‐Sabo et al. 2023 [82]	Experimental (clinical trial)	646 (NR)	Elementary education	Income: low income	United States	High income	Attitudinal and procedural
Cunningham‐Sabo et al. 2023 [83]	Experimental (clinical trial)	1426 (50.2)	Elementary education	Income: low income	United States	High income	Attitudinal e procedural
Labbé et al. 2023 [84]	Quasi‐experimental (before and after)	149 (50)	Elementary education	NR	Canada	High income	Attitudinal, conceptual and procedural
Vandyousefi et al. 2023 [85]	Quasi‐experimental (before and after)	2414 (53.2)	Elementary education	Income: low income Etnia: minorias raciais	United States	High income	Anthropometric and attitudinal
Hudson et al. 2023 [86]	Quasi‐experimental (before and after)	895 (53.2)	Elementary education	Ethnicity: Hispanic	United States	High income	Attitudinal
Teshome et al. 2023 [87]	Quasi‐experimental (before and after)	41 (34)	High school education	NR	Canada	High income	Attitudinal and conceptual
Muzaffar et al. 2024 [88]	Quasi‐experimental (before and after)	178 (80)	Elementary education	NR	United States	High income	Anthropometric, attitudinal and procedural

Abbreviation: NR, Not reported.

Thirty‐five studies (63.6%) reported equity‐related recruitment criteria, most commonly targeting low‐income or socioeconomically disadvantaged populations (*n* = 21; 60%). Although all studies were conducted in high‐income countries, mainly the United States (*n* = 26; 47.3%), the United Kingdom (*n* = 7; 12.7%), and Canada (*n* = 5; 9.1%) (Figure S1), many targeted socially or economically disadvantaged populations. Reported outcomes were most frequently attitudinal (*n* = 48; 87.3%) and procedural (*n* = 33; 60%). For example, attitudinal outcomes included children's attitudes toward food, food preferences, and willingness to try fruits and vegetables, as assessed in interventions such as Texas Sprouts [9, 67, 72, 74, 79, 81, 85, 86]. Procedural outcomes commonly included cooking and food preparation skills, such as recipe interpretation, food handling, and culinary techniques, as observed in programs such as Cooking With Kids [39, 40, 45, 48].

### 3.3 Characteristics of Culinary Practice Interventions

Among the 55 studies, 39 distinct interventions were identified (Table 2). Texas Sprouts was the most frequently reported (*n* = 8; 14.5%) [9, 67, 72, 74, 79, 81, 85, 86]. Theoretical frameworks were cited in 20% of studies, predominantly the Social Cognitive Theory (*n* = 11).

**TABLE 2:** Characteristics of culinary practice interventions for children and adolescents in school settings (*n* = 39 interventions).

Intervention name (Acronym)	Program objective	Number of sessions and workload (min)	Program's theoretical approach	Year of first published study
Cookshops (CS) [37]	Combination of chronic disease prevention and promotion of sustainable food choices in a program for elementary school children from low‐income urban areas.	10 (75)	Social Cognitive Theory	1998
Health Education Intervention for Taste Development [38]	Promotion of dish selection, a farming experience, a cooking experience, and a lecture on digestive absorption for elementary school children.	3 (600)	Experiential learning	2008
Cooking With Kids [39, 40, 45, 48]	Exploration of healthy foods with the five senses, acquisition of practical preparation skills, enjoyment in a collaborative environment and exercise of freedom of choice.	11 (60)	NR	2009
Stephanie Alexander Kitchen Garden Program (SAKG) [44, 46]	Increase children's appreciation and enjoyment of fresh and seasonal foods; develop skills, knowledge, and confidence in the kitchen and garden; promote understanding of environmental sustainability; create a learning environment that enhances self‐esteem and positive relationships.	NR (90)	NR	2012
Intervention Program to Encourage High School Students to Eat Lunch at School [42]	Significantly increase: (a) the average number of days high school students stay at school for lunch and (b) the proportion of students who stay at school for lunch every day.	NR (NR)	Theory of Planned Behavior	2012
Chefs Adopt a School Scheme (CAAS) [43]	Teaching children about food, food origins, healthy eating and food preparation.	3 (NR)	NR	2013
Multifactorial Intervention for Hispanic Children at High Risk of Obesity [47]	Reduction of body mass index in six‐ to eight‐year‐old children at high risk of obesity through a multicomponent nutritional intervention.	4 (150)	NR	2014
Cooking and Active Leisure Tu y Alícia por la Salud (CAL‐TAS) [49]	Improvement of eating habits and promotion of activities among adolescents.	7 (90)	NR	2014
Food, Math, and Science Teaching Enhancement Resource (FoodMASTER) [50]	Use of food as a tool for teaching mathematics and science.	24 (45)	NR	2015
Los Angeles Sprouts (LA Sprouts) [10, 11, 54]	Improvement of dietary intake and reduction of obesity among students in grades 3 to 5 (8–12 years).	12 (45)	NR	2015
Cooking Program For Alternative Education Students [51]	Active engagement of youth in meal preparation and sharing in a positive, supportive manner, promoting the learning of new skills and strengthening bonds between students and adults during sessions.	6 (NR)	Social–Environmental Learning Positive Youth Development	2015
Kids in the Kitchen [52]	Exposure of young children to a variety of fruits and vegetables, providing opportunities to prepare and taste foods, without focusing on the amount consumed.	10 (90)	NR	2015
Jamie Oliver's Kitchen Garden Project (JOKGP) [53, 58]	Promotion of children's culinary skills, willingness to try new foods, and better understanding of food origins (from farm to table).	NR (90)	NR	2015
Common Threads [55]	Primary focus on culinary skills, using hands‐on instruction to teach nutrition and promote cultural awareness.	10 (120)	NR	2016
The Spice MyPlate [56]	Motivation of African American students to achieve improvements in diet quality.	6 (120)	NR	2016
Taste Lessons (TL) [57]	Increase children's interest in food and expand their knowledge and skills regarding healthy and mindful eating behavior.	1 (45)	Ajzen's Theory of Planned Behavior	2016
Teen Cuisine [59]	Teaching youth important food preparation and cooking skills, as well as healthy eating patterns for obesity prevention.	6 (90)	Transactional Socioecological	2017
Cook Healthy Edible Food (CHEF) [60]	Teaching food safety, proper knife use and preparation of meals and snacks from scratch in a program offered by chefs in public schools.	5 (150)	NR	2017
Cooking Bus [61]	Teaching culinary skills, healthy eating, food safety, and hygiene; making cooking enjoyable; raising awareness about the importance of good nutrition and consumption of fresh ingredients; guiding parents on how to obtain local foods at low cost; emphasizing the role of fruits and vegetables in children's diets; motivating teachers, assistants, parents, and children; empowering teachers to teach cooking with confidence; valuing food education in schools and communities in Wales; providing hands‐on cooking experiences and raising awareness about food origins.	NR (90)	NR	2017
Choose Health: Food, Fun, and Fitness (CHFFF) [62]	Enhancement of evidence‐based behaviors for the prevention of obesity and chronic diseases, such as increasing the intake of vegetables, fruits and whole grains, reducing sugar‐sweetened beverages and foods high in fat and sugar and promoting active play.	6 (67.5)	Experiential Learning Social Cognitive Theory	2018
iGrow (iGrow) [63]	Enhancement of gardening skills, culinary competence, and family mealtime for early adolescents and their caregivers.	5 (120)	Social Cognitive Theory	2018
Waves [64]	Increase children's physical activity levels at school and home and support the development of healthy behavior skills in families through activity‐based learning.	12 (60)	NR	2018
Veggiecation [65]	Improvement of children's acceptance and consumption of vegetables through interactive learning and engagement.	4 (40)	NR	2018
Nutritional Intervention for Children With Mental and Neurodevelopmental Disorders [66]	Promotion of a weekly experience of cooking from scratch, eating the food, sampling each other's dishes, sharing with staff and school peers, and taking some home.	NR (NR)	NR	2019
Texas Sprouts (TX Sprouts) [9, 67, 72, 74, 79, 81, 85, 86]	Improvement of psychosocial constructs related to diet, such as knowledge in nutrition, gardening, and cooking, self‐efficacy, attitudes, and children's willingness to try and prefer fruits, vegetables, and legumes (FVL); reduction of food insecurity through parents, aiming to increase FVL intake and reduce obesity and blood pressure.	11 (60)	NR	2019
Exercício Gyotaku [68]	Exploration, through intervention, of the potential of a sensory experiential activity in a school setting ‐ with a focus on cooking and tactile play—to promote fish acceptance among 11‐ to 13‐year‐old children.	NR(NR)	NR	2020
Culinary Education Program for Schoolchildren [69]	Promotion of healthy eating behaviors using cooking as an educational tool.	30 (60)	NR	2020
Garden‐Based Integrated Intervention [70]	Improvement of children's eating behavior regarding vegetables.	12 (40)	Social Cognitive Theory	2020
Practice‐Focused Nutrition Intervention in Hungarian [71]	Improvement of children's weight, knowledge, and lifestyle, involving the family.	5 (90)	NR	2020
EgizuSUK! Project [73]	Reduction of food neophobia and promotion of behavior change regarding vegetables through cooking classes.	3 (60)	NR	2021
School Cooking Program in Japan [75]	Encouragement of children's participation in home cooking.	3 (45)	Social Cognitive Theory	2021
KidChen [76]	Provision of first‐hand experience for children in preparing their own healthy meals, incorporating touch, sight, taste, hearing, and smell.	NR (NR)	Social Cognitive Theory Experiential learning	2022
Professional Cooking (PC) [77]	Measuring and weighing ingredients, reading and following recipes, applying various food preparation, cooking, and assembly techniques, as well as preparing meals.	81 (65)	NR	2022
Food Camp [78]	Encouragement of a healthier lifestyle through students' experiences with natural products, cooking, learning, and camping activities.	5 (NR)	NR	2022
Cooking Kit for Kids (COOKKIT) [80]	Engagement of low‐income families through school‐based cooking and nutrition classes, with meal kits and shopping lists to transfer and sustain food skills at home.	4 (90)	NR	2023
Fuel for Fun (FFF) [82, 83]	Improvement of the nation's nutrition and health by promoting healthy eating and physical activity habits in children.	5 (120)	Social Cognitive Theory Experiential learning	2023
Apprenti en Action [84]	Teaching students to read and interpret recipes, apply various culinary techniques in food preparation, adopt food safety practices, and introduce basic concepts of healthy eating, nutrition, and cooking.	6 (45)	NR	2023
Pulses Make Perfect Sense (PMPS) [87]	Targets food literacy, including knowledge of food and nutrition, food skills, self‐efficacy and confidence, food choices, and ecological (i.e., external) factors.	7 (52.5)	Food literacy	2023
Coordinated Approach to Child's Health (CATCH) [88]	Improvement of children's nutrition and physical activity behaviors by promoting healthy eating, increasing physical activity, and reducing screen time to combat childhood obesity.	6 (25)	NR	2024

Abbreviation: NR, Not reported.

Nearly half of the studies (*n* = 26; 47.3%) integrated culinary activities into core subjects such as mathematics, science, or language. Food was addressed across psychosocial, socio‐environmental, and sociocultural dimensions, with psychosocial aspects being most prevalent (*n* = 48; 87.3%) (Figure 2A). Psychosocial dimensions commonly focused on food‐related knowledge, attitudes, self‐efficacy, and willingness to try new foods, as observed in interventions such as Texas Sprouts [9, 67, 72, 74, 79, 81, 85, 86] and Taste Lessons [57]. Socio‐environmental dimensions addressed factors influencing food choices and behaviors in family, school, and community settings, exemplified by Cooking Kit for Kids (COOKKIT) [80], which sought to extend cooking practices into the home, and Cooking Bus [61], which engaged schools, families, and communities. Sociocultural dimensions emphasized cultural meanings and social experiences related to food, illustrated by Common Threads [55], which combined culinary education with cultural awareness, and the Stephanie Alexander Kitchen Garden Program [44, 46], which promoted appreciation of seasonal foods and food culture.

**FIGURE 2:**
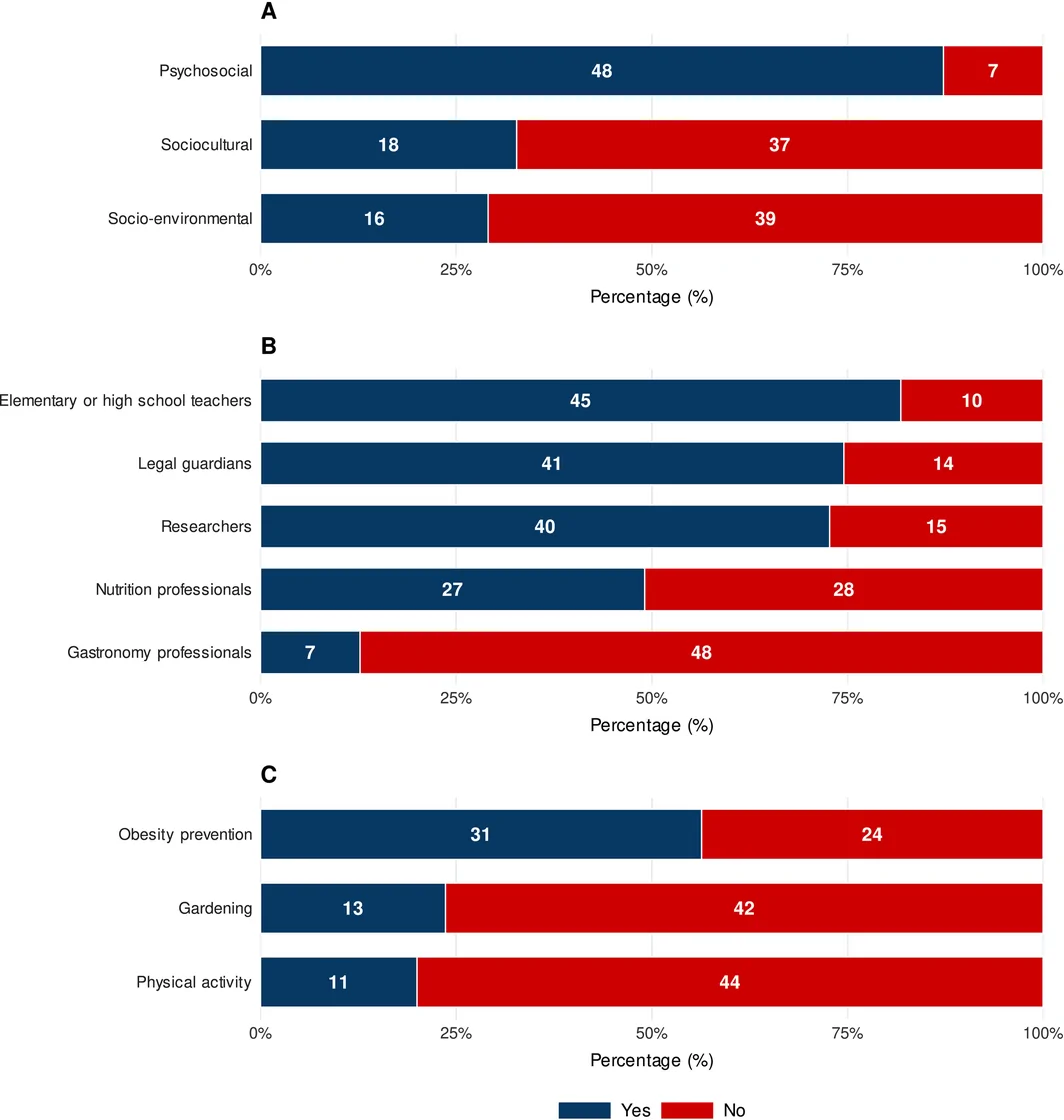
Aspects of food, actors involved and other approaches (gardening, physical education and obesity prevention) in culinary interventions for children and adolescents in a school environment (*n* = 55 studies). (A) Psychosocial, socio‐environmental and sociocultural aspects of food considered in culinary practice interventions for children and adolescents in a school environment (*n* = 55 studies). (B) Actors involved in the development and implementation of culinary practice interventions for children and adolescents in a school environment (*n* = 55 studies). (C) Cooking interventions for children and adolescents in school settings that combined other approaches such as gardening, physical activity, and obesity prevention (*n* = 55 studies).

Interventions were mainly implemented by teachers (*n* = 45; 81.8%), researchers (*n* = 40; 72.7%), and legal guardians (*n* = 41; 74.5%). Nutrition professionals participated in 27 studies (49.1%), while gastronomy professionals appeared in only seven (12.7%) (Figure 2B).

Gastronomy professionals first appeared in 2012 in the Intervention Program to Encourage High School Students to Eat Lunch at School [42], an initiative based on the Theory of Planned Behavior that encouraged students to remain at school for lunch rather than purchase food from nearby fast‐food restaurants by addressing attitudes, social influences, and perceived control over lunchtime choices. Many interventions also addressed obesity prevention (*n* = 31; 56.4%), gardening (*n* = 13; 23.6%), or physical activity (*n* = 11; 20%) (Figure 2C).

Forty‐seven studies (85.5%) reported recipes prepared by students. Across the 39 mapped interventions, the most frequently addressed food groups were fruits, vegetables, legumes, pulses, and nuts (*n* = 29; 52.7%); cereals, tubers, and roots (*n* = 27; 49.1%); meats, eggs, milk, and dairy products (*n* = 18; 32.7%); and oils and fats (*n* = 7; 12.7%) (Table 3). These frequencies refer to food groups included in the interventions, regardless of whether they were used in recipes or other culinary activities. All interventions prioritized unprocessed or minimally processed foods.

**TABLE 3:** Food groups used in preparations developed in culinary practice interventions for children and adolescents in school settings (*n* = 39 interventions).

Intervention name/Acronym (References)	Classification of culinary preparations by food groups				Culinary skills developed in the intervention
	Fruits, vegetables, legumes, pulses, and nuts (*n* = 29)	Cereals, tubers, and roots (*n* = 27)	Meat, poultry, fish, eggs, milk, and dairy products (*n* = 18)	Oils and fats (*n* = 7)
Cookshops (CS) [37]	Yes	Yes	No	No	Food handling and preparation techniques; food hygiene and safety practices.
Health Education Intervention for Taste Development [38]	Yes	Yes	Yes	No	NR
Cooking With Kids [39, 40, 45, 48]	Yes	Yes	No	Yes	NR
Stephanie Alexander Kitchen Garden Program (SAKG) [44, 46]	Yes	Yes	No	No	Food handling and preparation techniques
Intervention Program to Encourage High School Students to Eat Lunch at School [42]	Yes	Yes	Yes	No	NR
Chefs Adopt a School Scheme (CAAS) [43]	Yes	Yes	No	No	Food handling and preparation techniques; food hygiene and safety practices; sensory and physicochemical analysis of foods.
Multifactorial Intervention for Hispanic Children at High Risk of Obesity [47]	NR	NR	NR	NR	Food handling and preparation techniques; reading of labels, recipes, dietary guidelines, and food information.
Cooking and Active Leisure Tu y Alícia por la Salud (CAL‐TAS) [49]	Yes	No	No	No	NR
Food, Math, and Science Teaching Enhancement Resource (FoodMASTER) [50]	Yes	Yes	Yes	No	Food handling and preparation techniques; food hygiene and safety practices; sensory and physicochemical analysis of foods; reading of labels, recipes, dietary guidelines and food information.
Los Angeles Sprouts (LA Sprouts) [10, 11, 54]	Yes	Yes	Yes	Yes	Food handling and preparation techniques; food hygiene and safety practices; reading of labels, recipes, dietary guidelines, and food information; sensory and physicochemical analysis of foods.
Cooking Program for Alternative Education Students [51]	Yes	Yes	Yes	Yes	NR
Kids in the Kitchen [52]	NR	NR	NR	NR	Food handling and preparation techniques; reading of labels, recipes, dietary guidelines, and food information.
Jamie Oliver's Kitchen Garden Project (JOKGP) [53, 58]	Yes	Yes	No	No	Food handling and preparation techniques; food hygiene and safety practices.
Common Threads [55]	NR	NR	NR	NR	Food handling and preparation techniques; food hygiene and safety practices; reading of labels, recipes, dietary guidelines, and food information.
The Spice MyPlate [56]	Yes	Yes	Yes	No	Food handling and preparation techniques; food hygiene and safety practices; sensory and physicochemical analysis of foods.
Taste Lessons (TL) [57]	NR	NR	NR	NR	Food handling and preparation techniques; food hygiene and safety practices; reading of labels, recipes, dietary guidelines, and food information.
Teen Cuisine [59]	No	Yes	Yes	No	Food handling and preparation techniques; food hygiene and safety practices; reading of labels, recipes, dietary guidelines, and food information.
Cook Healthy Edible Food (CHEF) [60]	NR	NR	NR	NR	Food handling and preparation techniques; food hygiene and safety practices.
Cooking Bus [61]	NR	NR	NR	NR	Food handling and preparation techniques; food hygiene and safety practices; reading of labels, recipes, dietary guidelines, and food information.
Choose Health: Food, Fun, and Fitness (CHFFF) [62]	Yes	No	No	No	Reading of labels, recipes, dietary guidelines, and food information; sensory and physicochemical analysis of foods.
iGrow (iGrow) [63]	Yes	Yes	No	No	Food hygiene and safety practices.
Waves [64]	NR	NR	NR	NR	Food handling and preparation techniques.
Veggiecation [65]	Yes	Yes	No	No	Food handling and preparation techniques.
Nutritional Intervention for Children With Mental and Neurodevelopmental Disorders [66]	Yes	Yes	Yes	No	Food handling and preparation techniques; food hygiene and safety practices.
Texas Sprouts (TX Sprouts) [9, 67, 72, 74, 79, 81, 85, 86]	Yes	Yes	No	No	Food hygiene and safety practices; reading of labels, recipes, dietary guidelines, and food information.
Exercício Gyotaku [68]	No	No	Yes	Yes	Food handling and preparation techniques.
Culinary Education Program for Schoolchildren [69]	Yes	Yes	Yes	No	Food handling and preparation techniques; food hygiene and safety practices.
Garden‐Based Integrated Intervention [70]	Yes	Yes	Yes	No	NR
Practice‐Focused Nutrition Intervention in Hungarian [71]	Yes	Yes	Yes	No	Food handling and preparation techniques; reading of labels, recipes, dietary guidelines, and food information.
EgizuSUK! Project [73]	Yes	Yes	No	No	Food handling and preparation techniques.
School Cooking Program in Japan [75]	Yes	No	Yes	No	Food handling and preparation techniques.
KidChen [76]	Yes	Yes	Yes	Yes	Food handling and preparation techniques; food hygiene and safety practices.
Professional Cooking (PC) [77]	NR	NR	NR	NR	Food handling and preparation techniques; food hygiene and safety practices; reading of labels, recipes, dietary guidelines, and food information.
Food Camp [78]	Yes	Yes	Yes	No	Food handling and preparation techniques; reading of labels, recipes, dietary guidelines, and food information.
Cooking Kit for Kids (COOKKIT) [80]	Yes	Yes	Yes	No	Food handling and preparation techniques; food hygiene and safety practices; sensory and physicochemical analysis of foods.
Fuel for Fun (FFF) [82, 83]	Yes	Yes	No	Yes	NR
Apprenti en Action [84]	Yes	Yes	Yes	Yes	Food handling and preparation techniques; reading of labels, recipes, dietary guidelines and food information.
Pulses Make Perfect Sense (PMPS) [87]	Yes	Yes	Yes	No	Food handling and preparation techniques; food hygiene and safety practices; reading of labels, recipes, dietary guidelines, and food information.
Coordinated Approach to Child's Health (CATCH) [88]	Yes	Yes	No	No	NR

Abbreviation: NR, Not reported.

Regarding culinary skills, the most frequently addressed category was food handling and preparation techniques (*n* = 28; 71.8%), including measuring ingredients, following recipes, and applying cooking methods, as reported in interventions such as Professional Cooking [77]. Hygiene and food safety practices (*n* = 19; 46.2%) were emphasized in programs such as CHEF [60] and Cooking Bus [61]. Label reading, recipes, dietary guidelines, and food information (*n* = 12; 30.8%) were addressed in interventions such as Apprenti en Action [84] and Pulses Make Perfect Sense [87]. Sensory and physicochemical food analysis (*n* = 6; 15.4%) included activities focused on taste, smell, texture, and food exploration, as observed in KidChen [76] and the Health Education Intervention for Taste Development [38]. Details on recipes and culinary skills developed in the interventions can be found in the Supporting Information (Table S5).

## 4 Discussion

### 4.1 Main Finding

This scoping review mapped 39 school‐based culinary interventions described across 55 studies involving children and adolescents. Most interventions were conducted in high‐income countries, frequently targeted socioeconomically disadvantaged populations, and primarily involved elementary school students. Culinary interventions were commonly based on Social Cognitive Theory and implemented in collaboration with teachers, with limited involvement of gastronomy professionals. Integrated into core curriculum subjects, they addressed multiple dimensions of food—psychosocial, socio‐environmental, and sociocultural—although psychosocial aspects predominated. Interventions often incorporated gardening, physical activity, and obesity prevention components, while recipes emphasized fruits, vegetables, legumes, pulses, nuts, and other minimally processed foods. The outcomes most frequently assessed were attitudinal and procedural, and the culinary skills developed mainly related to food handling and preparation techniques.

### 4.2 Comparison With Other Reviews

The studies included in this review predominantly used randomized and quasi‐experimental designs, consistent with a previous review (1998–2020) that identified quantitative approaches as the primary method for evaluating culinary interventions for children and adolescents [89]. That review also highlighted the scarcity of qualitative research on social representations, implementation processes, and stakeholder perspectives [89].

The predominance of quantitative methods may limit understanding of the relational and contextual dimensions of culinary practices, which are not easily captured by standardized outcomes. Future research could benefit from greater use of qualitative approaches, including participant observation, ethnography, and semi‐structured interviews, to explore how culinary interventions are experienced and integrated into educational settings [90].

Although no age limits were imposed, most school‐based culinary interventions targeted elementary students, suggesting that this stage is strategic for fostering healthy eating habits, as children are developing food preferences and may be more receptive to practical and playful activities [91]. A previous review of children's cooking programs similarly reported heterogeneity in age groups, outcomes, and intervention characteristics, identifying younger children as the main target audience despite challenges in engaging this population [22].

Previous reviews show that nutritional interventions for children and adolescents are predominantly conducted in high‐income countries, consistent with our findings [92, 93, 94]. This concentration limits the applicability of evidence to middle‐ and low‐income settings, where social determinants of health and school infrastructure may substantially affect implementation [95].

Most studies reported equity‐related recruitment criteria, mainly targeting low‐income or socioeconomically disadvantaged populations. However, race and ethnicity, gender, geographic location, and special educational needs were less frequently reported, despite their relevance to culinary interventions for young populations.

A few initiatives involved children with mental or neurodevelopmental disorders [66] and African American and Hispanic groups [56], but these remain limited. A review on nutritional strategies for Indigenous youth likewise highlighted the scarcity of studies in this population, although culturally adapted, participatory, and visibility‐oriented programs were associated with better outcomes [96].

In addition, although evidence from low‐ and middle‐income countries was limited, school‐based culinary interventions may be particularly relevant in these contexts, given the greater burden of food insecurity and structural socioeconomic constraints shaping children's dietary practices and school food environments [96].

The most frequently studied intervention was Texas Sprouts (TX Sprouts), likely due to its methodological rigor and comprehensive approach, combining culinary, gardening, and nutrition education activities in public schools, particularly among low‐income and Latino populations in the United States [67]. TX Sprouts exemplifies comprehensive school‐based culinary interventions through integration across educational domains, engagement with school communities, and implementation in vulnerable contexts. These characteristics may inform the design of interventions combining culinary practice with broader food and nutrition education strategies [9, 67, 72, 74, 79, 81, 85, 86]. Previous reviews have also identified TX Sprouts as a promising program [93, 97].

Consistent with a previous scoping review, Social Cognitive Theory was the main framework used in the analyzed interventions [18]. This theory emphasizes interactions among personal, behavioral, and environmental factors [98], supporting behavior change through shared experiences. However, its focus on habit modification through psychosocial measures may overlook everyday and contextual dimensions of food [99]. Our findings reflect this emphasis, with psychosocial dimensions predominating over socio‐environmental and sociocultural aspects.

The focus on psychosocial aspects suggests a tendency to understand food mainly through individual and interpersonal factors, such as attitudes, preferences, self‐efficacy, and eating behavior [100]. While relevant, this perspective may oversimplify food by underrepresenting socio‐environmental and sociocultural influences on children's dietary practices, including food availability, school policies, cultural norms, and family traditions [100]. These findings underscore the importance of integrating less‐explored dimensions to enhance students' critical thinking, engagement, agency, and socially responsible decision‐making, which are essential for exercising citizenship [101, 102].

This review found attitudinal outcomes to be the most common among the included studies, followed by procedural outcomes. This pattern reflects the interventions' aim to foster dietary habit changes through practical culinary experiences. The results align with prior research using culinary practices for obesity prevention, highlighting a behavior‐focused approach [103, 104]. Despite the relevance of attitudinal and procedural outcomes, the conceptual dimension of food was seldom addressed. Comprehensive food education programs should integrate conceptual, procedural, and attitudinal dimensions in a complementary and interconnected way to support meaning‐making and knowledge consolidation [103, 104].

Approximately half of the included studies integrated culinary activities with core academic subjects, particularly mathematics, science, and language arts. This approach positions culinary practices as a cross‐curricular strategy that supports academic learning while developing practical skills [105]. Food preparation was used to teach mathematical concepts, such as measurement and proportion [46, 50], and science topics related to chemical transformations, hygiene, food safety, and nutrients [41, 61]. Integrating culinary activities with other subjects may also strengthen students' understanding of the cultural, social, and environmental dimensions of food [106, 107].

The recipes in mapped interventions prioritized fruits, vegetables, legumes, pulses, and nuts, consistent with other reviews and dietary guidelines. This emphasis is appropriate given low consumption of plant‐based foods among children and adolescents and global recommendations to increase their intake for obesity and diet‐related disease prevention [53, 73]. Rather than replacing this focus, future strategies could broaden culinary education by incorporating other food groups relevant to local dietary patterns, while strengthening connections between culinary practices and broader social, political, and economic determinants of food choice and access [28].

Most interventions involved the participation of legal guardians. This inclusion is justified because children are protected subjects, and guardian involvement is crucial to achieving the goal of improving dietary habits, which largely reflects family practices [108]. Family participation may also strengthen consistency between home and school food environments. Tensions regarding the respective roles of families and schools in food education have been documented, with some stakeholders viewing healthy eating as primarily a parental responsibility while others emphasizing the school's role in promoting health [6]. This highlights the importance of strategies that foster shared engagement and responsibility for children's food education [63].

Regarding the composition of the teams implementing the interventions, teachers and other school staff predominated, with some participation from nutritionists and very limited involvement of gastronomy professionals. This may reflect the field's historical market orientation and the still‐limited integration of gastronomy professionals into scientific research [109]. However, gastronomy is gaining recognition as a scientific field [110, 111].

Only three studies included multidisciplinary teams integrating professionals from Education, Gastronomy, and Nutrition. Such collaboration can support more comprehensive food education by integrating pedagogical, nutritional, cultural, and culinary perspectives to address the complexity of food‐related issues [28]. Its limited use highlights opportunities to strengthen collaboration among education, nutrition, and gastronomy professionals in future school‐based culinary interventions [105, 106].

Regarding curricular content involving culinary skills, there was a clear emphasis on food handling and preparation techniques (e.g., knife skills, cooking methods, and cutting techniques). Developing these skills promotes culinary autonomy and facilitates interaction with food, contributing to health and psychosocial well‐being [22, 112].

In contrast, competencies such as label reading and sensory analysis appeared less frequently across interventions. These skills may contribute to food literacy by supporting a more critical understanding of food products and choices, particularly in contemporary food environments characterized by the widespread availability of ultra‐processed products [113]. The limited incorporation of sensory analysis activities, including the evaluation of taste, smell, texture, appearance, and physicochemical food quality, also represents an underexplored opportunity to connect culinary practice with scientific knowledge and strengthen interdisciplinarity with fields such as chemistry and biology.

### 4.3 Implications for School Health Policy, Practice, and Equity

This review highlights the potential of school‐based culinary interventions to promote food learning by integrating conceptual knowledge, practical skills, and attitudinal dimensions. Beyond developing cooking competencies, these interventions may foster critical engagement with food‐related issues and strengthen students' relationship with food. Schools provide a strategic setting for meaningful food education experiences, particularly for students with fewer opportunities to cook, taste, or learn about food outside school. Culinary education may therefore help reduce inequities in access to food‐related knowledge and skills [9, 10, 11].

The findings suggest opportunities to broaden the educational scope of culinary interventions by incorporating broader dimensions of food literacy, including food systems, food environments, sensory exploration, food culture, and critical engagement with food‐related issues. This may support more comprehensive food education and better address contemporary food challenges [16, 17].

The predominance of studies from high‐income countries highlights an important geographical gap. Future research should prioritize low‐ and middle‐income countries to inform contextually relevant policies and practices across diverse cultural, educational, and resource‐constrained settings [19, 85].

Given the predominance of quantitative research, future qualitative studies could explore perceptions, meanings, implementation processes, and contextual factors to inform more responsive and sustainable programs [40, 68, 80].

The review identified opportunities to strengthen links between culinary interventions and curricular objectives. Integrating culinary activities with curricular content may enhance their educational value across science, health, social studies, and environmental education. Socioscientific approaches could further support critical thinking and informed decision‐making by engaging students with issues such as food insecurity, dietary diversity, food systems, sustainability, and ultra‐processed foods [15, 16, 17].

Finally, few studies reported multidisciplinary teams involving professionals from Education, Nutrition, and Gastronomy. Future interventions could benefit from greater interdisciplinary collaboration integrating pedagogical, nutritional, cultural, sensory, and practical perspectives [17].

### 4.4 Strengths and Limitations

This scoping review provides a robust mapping of school‐based culinary interventions involving children and adolescents, conducted according to international standards and supported by a comprehensive search strategy. Categories derived from education and equity literature helped identify gaps and inform future research and educational policies. The active involvement of Gastronomy and Nutrition professionals (BB and LFT) also strengthened study identification and retrieval.

The limitations of this review mainly relate to the reporting of the included studies. Recipes, implementation procedures, intervention duration, and specific culinary skills were often insufficiently described, complicating data extraction and results presentation. To address these limitations, all review stages were conducted independently by two reviewers, with additional documents and protocols consulted when necessary to ensure consistency and completeness.

## 5 Conclusion

This scoping review found that school‐based culinary interventions for children and adolescents were predominantly conducted in high‐income countries using quantitative designs. Most studies addressed at least one equity dimension, primarily socioeconomic status, and focused on elementary school students. Interventions mainly evaluated eating habits, food choices, and dietary behaviors, with a predominant emphasis on obesity prevention and diet quality rather than critical engagement with broader food‐related issues.

These findings highlight important gaps and opportunities for future research, particularly in low‐ and middle‐income countries, through greater use of qualitative approaches, stronger multidisciplinary collaboration, and broader food education that incorporates sociocultural, socioenvironmental, and equity perspectives. Integrating food, equity, and disciplinary content in basic education may strengthen comprehensive food education, with gastronomy professionals contributing expertise that recognizes food as both a nutritional and sociocultural practice.

## Ethics Statement

Preparation of this paper did not involve primary research or data collection involving human subjects, and therefore, no institutional review board examination or approval was required.

## Conflicts of Interest

The authors declare no conflicts of interest.

## Supporting information


**Table S1:** Preferred reporting items for systematic reviews and meta‐analyses extension for scoping reviews (PRISMA‐ScR) checklist.
**Table S2:** Search strategy.
**Table S3:** Description of extracted data.
**Table S4:** Reasons for exclusion of studies assessed in full text.
**Figure S1:** Distribution of countries by studies on culinary interventions for children and adolescents in school settings (*n* = 55 studies).
**Table S5:** Recipes, techniques, and culinary skills developed in school‐based cooking practice interventions for children and adolescents (*n* = 39 interventions).

## Data Availability

The data that supports the findings of this study are available in the Supporting Information of this article.
