# Supplementary material for: Scoping Review of Culinary Interventions in Schools: Mapping Practices to Promote Food Education and Equity
Source: J Sch Health. 2026 Oct 15;96(10):e70243. doi: 10.1111/josh.70243 (PMC13671095; doi:10.1111/josh.70243)
Supplement: Supplementary file 1 [file JOSH-96-0-s001.docx]

Table S1. Preferred Reporting Items for Systematic reviews and Meta-Analyses extension for Scoping Reviews (PRISMA-ScR) Checklist

| **SECTION** | **ITEM** | **PRISMA-ScR CHECKLIST ITEM** | **REPORTED ON PAGE #** |
| --- | --- | --- | --- |
| **TITLE** | | | |
| Title | 1 | Identify the report as a scoping review. | 1 |
| **ABSTRACT** | | | |
| Structured summary | 2 | Provide a structured summary that includes (as applicable): background, objectives, eligibility criteria, sources of evidence, charting methods, results, and conclusions that relate to the review questions and objectives. | 1 |
| **INTRODUCTION** | | | |
| Rationale | 3 | Describe the rationale for the review in the context of what is already known. Explain why the review questions/objectives lend themselves to a scoping review approach. | 2-4 |
| Objectives | 4 | Provide an explicit statement of the questions and objectives being addressed with reference to their key elements (e.g., population or participants, concepts, and context) or other relevant key elements used to conceptualize the review questions and/or objectives. | 4 |
| **METHODS** | | | |
| Protocol and registration | 5 | Indicate whether a review protocol exists; state if and where it can be accessed (e.g., a Web address); and if available, provide registration information, including the registration number. | 4 |
| Eligibility criteria | 6 | Specify characteristics of the sources of evidence used as eligibility criteria (e.g., years considered, language, and publication status), and provide a rationale. | 4-5 |
| Information sources* | 7 | Describe all information sources in the search (e.g., databases with dates of coverage and contact with authors to identify additional sources), as well as the date the most recent search was executed. | 5 |
| Search | 8 | Present the full electronic search strategy for at least 1 database, including any limits used, such that it could be repeated. | 5 and Table S2 |
| Selection of sources of evidence† | 9 | State the process for selecting sources of evidence (i.e., screening and eligibility) included in the scoping review. | 5-6 |
| Data charting process‡ | 10 | Describe the methods of charting data from the included sources of evidence (e.g., calibrated forms or forms that have been tested by the team before their use, and whether data charting was done independently or in duplicate) and any processes for obtaining and confirming data from investigators. | 6-7 |
| Data items | 11 | List and define all variables for which data were sought and any assumptions and simplifications made. | 6-7 |
| Critical appraisal of individual sources of evidence§ | 12 | If done, provide a rationale for conducting a critical appraisal of included sources of evidence; describe the methods used and how this information was used in any data synthesis (if appropriate). | N/A |
| Synthesis of results | 13 | Describe the methods of handling and summarizing the data that were charted. | 7 |
| **RESULTS** | | | |
| Selection of sources of evidence | 14 | Give numbers of sources of evidence screened, assessed for eligibility, and included in the review, with reasons for exclusions at each stage, ideally using a flow diagram. | 7 |
| Characteristics of sources of evidence | 15 | For each source of evidence, present characteristics for which data were charted and provide the citations. | 8-9 |
| Critical appraisal within sources of evidence | 16 | If done, present data on critical appraisal of included sources of evidence (see item 12). | N/A |
| Results of individual sources of evidence | 17 | For each included source of evidence, present the relevant data that were charted that relate to the review questions and objectives. | 8-9 |
| Synthesis of results | 18 | Summarize and/or present the charting results as they relate to the review questions and objectives. | 8-9 |
| **DISCUSSION** | | | |
| Summary of evidence | 19 | Summarize the main results (including an overview of concepts, themes, and types of evidence available), link to the review questions and objectives, and consider the relevance to key groups. | 10 |
| Limitations | 20 | Discuss the limitations of the scoping review process. | 17 |
| Conclusions | 21 | Provide a general interpretation of the results with respect to the review questions and objectives, as well as potential implications and/or next steps. | 17-18 |
| **FUNDING** | | | |
| Funding | 22 | Describe sources of funding for the included sources of evidence, as well as sources of funding for the scoping review. Describe the role of the funders of the scoping review. | Title Page |

JBI = Joanna Briggs Institute; PRISMA-ScR = Preferred Reporting Items for Systematic reviews and Meta-Analyses extension for Scoping Reviews.

* Where *sources of evidence* (see second footnote) are compiled from, such as bibliographic databases, social media platforms, and Web sites.

† A more inclusive/heterogeneous term used to account for the different types of evidence or data sources (e.g., quantitative and/or qualitative research, expert opinion, and policy documents) that may be eligible in a scoping review as opposed to only studies. This is not to be confused with *information sources* (see first footnote).

‡ The frameworks by Arksey and O’Malley (6) and Levac and colleagues (7) and the JBI guidance (4, 5) refer to the process of data extraction in a scoping review as data charting*.*

§ The process of systematically examining research evidence to assess its validity, results, and relevance before using it to inform a decision. This term is used for items 12 and 19 instead of "risk of bias" (which is more applicable to systematic reviews of interventions) to include and acknowledge the various sources of evidence that may be used in a scoping review (e.g., quantitative and/or qualitative research, expert opinion, and policy document).

*From:* Tricco AC, Lillie E, Zarin W, O'Brien KK, Colquhoun H, Levac D, et al. PRISMA Extension for Scoping Reviews (PRISMAScR): Checklist and Explanation. Ann Intern Med. 2018;169:467–473. [doi: 10.7326/M18-0850](http://annals.org/aim/fullarticle/2700389/prisma-extension-scoping-reviews-prisma-scr-checklist-explanation).

Table S2. Search Strategy

| **Base** | **Strategy** | **Results** |
| --- | --- | --- |
| Medline | (Child[MH] OR Child*[TIAB] OR Adolescent[MH] OR Adolescent*[TIAB] OR Adolescence[TIAB] OR Youth*[TIAB] OR Teen*[TIAB] OR Teenager*[TIAB] OR Students[MH] OR Student*[TIAB]) AND (Cooking[MH] OR Cooking[TIAB] OR Gastronomy[TIAB] OR "cooking skills"[TIAB] OR "culinary practices"[TIAB] OR cook*[TIAB] OR “culinary education”[TIAB] OR “culinary skills”[TIAB] OR Food and Nutrition Education[MH]) AND (Schools[MH] OR School*[TIAB] OR Education[MH] OR “Educational Activities”[TIAB] OR “Activities, Educational”[TIAB] OR “Activity, Educational”[TIAB] OR “Educational Activity”[TIAB] OR “Literacy Programs”[TIAB] OR “Literacy Program”[TIAB] OR “Program, Literacy”[TIAB] OR “Programs, Literacy”[TIAB] OR Training[TIAB] OR “Training Programs”[TIAB] OR “Programs, Training”[TIAB] OR “Program, Training”[TIAB] OR “Training Program”[TIAB] OR Workshop*[TIAB] OR Teaching[MH]) | 2655 |
| Virtual Health Library Portal | (MH:Child OR MH:Adolescent OR TW:Adolescence OR TW:Youth* OR TW:Teen* OR MH:Students) AND (MH:Cooking* OR TW:Gastronomy OR TW:"Culinary practices" OR TW:“culinary education” OR TW:“culinary skills” OR MH:Food and Nutrition Education) AND (TW:Educ* OR MH:Schools OR TW:Training OR TW:Workshop*) | 423 |
| ERIC | Descriptor:child* OR adolescent* OR Student* AND “primary schools” OR “high schools” OR “junior high schools” AND "Cooking Instruction" OR gastronomy OR "cooking skills" OR "culinary practices" OR cook OR "culinary education" OR "culinary skills" OR “Food and Nutrition Education” AND Education OR “Educational Activities” OR “Activities, Educational” OR “Activity, Educational” OR “Educational Activity” OR “Literacy Programs” OR “Literacy Program” OR “Program, Literacy” OR “Programs, Literacy” OR Training OR “Training Programs” OR “Programs, Training” OR “Program, Training” OR “Training Program” OR Workshop* OR Teaching | 25 |
| Scopus | TITLE-ABS-KEY((child* OR adolescent* OR Student*) AND (“primary schools” OR “high schools” OR “junior high schools”)) AND TITLE-ABS-KEY("Cooking Instruction" OR gastronomy OR "cooking skills" OR "culinary practices" OR cook OR "culinary education" OR "culinary skills" OR “Food and Nutrition Education”)AND TITLE-ABS-KEY(Education OR “Educational Activities” OR “Activities, Educational” OR “Activity, Educational” OR “Educational Activity” OR “Literacy Programs” OR “Literacy Program” OR “Program, Literacy” OR “Programs, Literacy” OR Training OR “Training Programs” OR “Programs, Training” OR “Program, Training” OR “Training Program” OR Workshop* OR Teaching) | 132 |
| Google Scholar | (Child* OR Adolescent* OR Adolescence OR Youth* OR Teen* OR Students) AND (Cooking* OR Gastronomy OR "Culinary practices" OR “culinary education” OR “culinary skills” OR “Food and Nutrition Education”) AND (Educ* OR Schools OR Training OR Workshop*) | 47600  (First 200 to be screened) |
| Total | 3,435 | |

**Table S3.** Description of extracted data

| Relevant information | Extracted variables |
| --- | --- |
| Study characteristics | author, year, country, region, income level, total number of children and adolescents participating, percentage of female participants, education level (elementary or secondary), age, and grade/year. The study approach (quantitative, qualitative, or mixed methods) and study type (cross-sectional, retrospective or prospective longitudinal, experimental, quasi-experimental, qualitative interviews, focus groups, or other) were also recorded. |
| culinary practice intervention characteristics. | intervention name and acronym, study-reported objectives and our interpretation of the information, theoretical learning approach, participants involved (e.g., gastronomy professionals, nutritionists, teachers, parents/caregivers, undergraduates, postgraduates, or researchers), and whether the intervention integrated culinary practices with any aspect of the basic education curriculum. Data were also extracted regarding curricular areas involved, inclusion of gardening, physical activity, or obesity prevention components, sociocultural, psychosocial, and socio-environmental aspects addressed, number of lessons, sessions or workshops, duration (minutes), intervention components and culinary skills developed, and recipes used. |

Table S4. Reasons for exclusion of studies assessed in full text

| Author  (year) | Title | Reasons for exclusion |
| --- | --- | --- |
| Karpouzis (2024) | Impact and process evaluation of a primary-school Food Education and Sustainability Training (FEAST) program in 10-12-year-old children in Australia: pragmatic cluster non-randomized controlled trial | Wrong context |
| Mekonnen (2023) | Evaluation of effectiveness of school-based nutrition education in improving the consumption of pulses-based food among female adolescents in Northwest Ethiopia: a cluster randomized controlled trial | Wrong concept |
| Luesse (2019) | In Defense of Food Curriculum: A Mixed Methods Outcome Evaluation in Afterschool | Wrong concept |
| Assis (2014) | Avaliação do conhecimento nutricional e comportamento alimentar após educação alimentar e nutricional em adolescentes de Juiz de Fora – MG | Wrong concept |
| Rangel (2014) | Ensinando e aprendendo sobre alimentação e nutrição através da educação em ciências: uma interseção de conhecimentos | Wrong population |
| Irala (2001) | Manual para Escolas: A Escola promovendo hábitos alimentares saudáveis | Wrong concept |
| Pivarnik (1994) | A food-safety curriculum fop sec Id.- and third-grade elementary students | Wrong concept |
| Cos (1992) | Consumo de alimentos comerciales en la infancia. Influencia de un programa de información en nutrición | Wrong concept |
| Coates (1985) | Modifying the Snack Food Consumption Patterns of Inner City High School Students: The Great Sensations Study’ | Wrong concept |
| Ramos (2019) | Educación para la salud. Nutrición y gastronomía en las ciudades autónomas de Melilla y Ceuta | Wrong concept |
| Farley (2019) | Cooking up a delicious experiential learning activity | Wrong population |
| Maldonado (2022) | A proposal for food and nutrition education integrated into the preschool and elementar school curriculum | Wrong population |
| Policastro (2023) | Healthy helpers: using culinary lessons to improve children’s culinary literacy and self-efficacy to cook | Wrong context |
| Oliveira (2022) | Interdisciplinary Educational Interventions Improve Knowledge of Eating, Nutrition, and Physical Activity of Elementary Students | Wrong concept |
| Isoldi (2020) | Cooking Up Energy: A Culinary Education Program for Children with Parent Outreach. | Data unavailable |
| Miller (2016) | “What’s Cooking?”: Qualitative Evaluation of a  Head Start Parent–Child Pilot Cooking Program | Wrong context |
| Antwi (2020) | Primary school-based nutrition education intervention on nutrition knowledge, attitude and practices among school-age children in Ghana | Wrong concept |
| Ahmadpour (2023) | Development and evaluation of an intervention to improve food and nutrition literacy among Iranian Kurdish primary school children: An application of intervention mapping approach | Wrong concept |
| Ahmadpour (2020) | Children Food and Nutrition Literacy - a New Challenge in Daily Health and Life, the New Solution: Using Intervention Mapping Model Through a Mixed Methods Protocol | Wrong concept |
| Wall (2012) | Nutrition Education Intervention Improves Vegetable-Related Attitude, Self-Efficacy,  Preference, and Knowledge of Fourth-Grade Students | Wrong concept |
| Gaspar (2016) | A new educational tool to learn about hydration: taste workshops for children | Wrong concept |
| Mogre (2024) | A school-based food and nutrition education intervention increases nutrition-related  knowledge and fruit consumption among primary school children in northern Ghana | Wrong concept |
| Oakley (2017) | Peer-Led Culinary Skills Intervention for Adolescents: Pilot Study of the Impact on Knowledge, Attitude, and Self-efficacy | Wrong context |
| Gatto (2016) | LA sprouts randomized controlled nutrition, cooking and gardening programme reduces obesity and metabolic risk in Hispanic/Latino youth | Wrong concept |
| Fierro (2019) | Efecto de un programa para profesores sobre cambio de hábitos alimentarios y habilidades culinarias en escuelas públicas de Chile | Wrong concept |
| Hagerdon (2018) | Teens Implementing a Childhood Obesity Prevention Program in the Community: Feasibility and Perceptions of a Partnership with HSTA and iCook 4-H | Wrong context |
| Allirot (2016) | Involving children in cooking activities: A potential strategy for directing food choices toward novel foods containing vegetables | Wrong context |
| Hume (2014) | Remote school gardens: exploring a cost-effective and novel way to engage Australian Indigenous students in nutrition and health | Wrong population |
| Froberg (2018) | Effects of an Empowerment-Based Health-Promotion School Intervention on Physical Activity and Sedentary Time among Adolescents in a Multicultural Area | Wrong concept |
| Chen (2014) | Cooking up diversity: Impact of a multi-component, multi-cultural, experiential intervention on food and cooking behaviors among elementary-school students from low-income ethnically diverse families | Wrong concept |
| Jaenke (2012) | The Impact of a School Garden and Cooking Program on Boys’ and Girls’ Fruit and Vegetable Preferences, Taste Rating, and Intake | Wrong concept |
| Carleton (1991) | The Pawtucket Heart Health Program. Influencing adolescent eating patterns. | Wrong concept |
| Strumpler (2014) | Changes in Fruit and Vegetable Consumption of Third-Grade Students in Body Quest: Food of the Warrior, a 17-Class Childhood Obesity Prevention Program | Wrong concept |
| Eto 2019) | 埼玉県坂戸市における全小・中学校共通実施の「坂戸食育プログラム」の成果と課題 | Wrong concept |
| Santos (2015) | Effectiveness of an educational intervention to reduce the consumption of high-calorie foods in public school children in Teresina, Piauí (Brazil) | Wrong context |
| Thonney (2006) | Cooking Up Fun! A Youth Development Strategy that Promotes Independent Food Skills | Wrong context |
| Cunha (2013) | Effectiveness of a Randomized School-Based Intervention Involving Families and Teachers to Prevent Excessive Weight Gain among Adolescents in Brazil | Wrong concept |
| Duffrin (2005) | Developing Food Products and Enthusiastic Learners | Wrong context |
| Neyens (2017) | Seeing is doing. The implicit effect of TV cooking shows on children's use of ingredients | Wrong concept |
| Rosario (2016) | Health Promotion Intervention to Improve Diet Quality in Children: A Randomized Trial | Wrong concept |
| Majid (2022) | Outcome Evaluation on Impact of the Nutrition Intervention among Adolescents: A Feasibility, Randomised Control Study from Myheart Beat (Malaysian Health and Adolescents Longitudinal Research Team—Behavioural Epidemiology and Trial) | Wrong concept |
| Xu (2020) | The effect of comprehensive intervention for childhood obesity on dietary diversity among younger children: Evidence from a school-based randomized controlled trial in China | Wrong concept |
| Kattelmann (2018) | The iCook 4-H Study: Report on Physical Activity and Sedentary Time in Youth Participating in a Multicomponent Program Promoting Family Cooking, Eating, and Playing Together | Wrong context |
| Roccaldo (2024) | Improvement of Adherence to the Mediterranean Diet through a Nutrition Education Teaching Pack for Teachers within the “School Fruit Scheme” Program: An Italian Long-Term Trial in School Children | Wrong concept |
| Santarossa (2015) | An evaluation of the Kinect-Ed presentation, a motivating nutrition and cooking intervention for young adolescents in grades 6–8 | Wrong concept |
| Branun (2024) | Promoting nutrition literacy in children: a case study of a community partnership between a university andan elementary school | Wrong concept |
| Rosário (2012) | The Impact of an Intervention Taught by Trained Teachers on Childhood Overweight | Wrong concept |
| Brennan (2021) | Food environment intervention improves food knowledge, wellbeing and dietary habits in primary school children: Project Daire, a randomised controlled, factorial design cluster trial | Wrong concept |
| Sandover (2015) | Experiential learning and the visceral practice of ‘healthy eating’ | Wrong concept |
| Martinez-Garcia (2016) | Actividades de educación alimentaria y nutricional en escolares de 3o de primaria en el Colegio Público “La Serranica” de Aspe (Alicante): Experiencia piloto | Wrong concept |
| Felix (2021) | Análise Qualitativa do Programa Crescer Saudável na Escola – Visão dos Profissionais de Saúde, da Comunidade Escolar e da Família | Wrong concept |
| Mukhina (2014) | BeHealthy Charities Aid Foundation Program, Russia: A Program Impact Pathways (PIP) analysis | Data unavailable |
| Bisset (2008) | Assessing the Impact of the Primary School-based Nutrition Intervention Petits cuistots –parents en réseaux | Data unavailable |
| Xu (2017) | Comprehensive school-based intervention to control overweight and obesity in China: a cluster randomized controlled trial | Wrong concept |
| Viodel (2011) | Prevención de la obesidad en escuelas básicas de peñalolén: componente alimentación y nutrición | Wrong concept |
| Ruiz (2021) | Development and Pilot Testing of a Food Literacy Curriculum for High School-Aged Adolescents | Wrong context |
| Griffin (2017) | Process evaluation results of a cluster randomised controlled childhood obesity prevention trial: the WAVES study | Wrong concept |
| Adab (2014) | Preventing childhood obesity, phase II feasibility study focusing on South Asians: BEACHeS | Wrong concept |
| Pierce (2017) | A summer health program for african-american high school students in baltimore, maryland: community partnership for integrative health | Wrong context |
| Cunningham-Sabo (2016) | Fuel for Fun: a cluster-randomized controlled study of cooking skills, eating behaviors, and physical activity of 4th graders and their families | Data unavailable |
| Korsten-Reck (2005) | Freiburg Intervention Trial for Obese Children (FITOC): results of a clinical observation study | Wrong concept |
| Coleman (2010) | Outcomes From a Culturally Tailored Diabetes Prevention Program in Hispanic Families From a Low-Income School | Wrong population |
| Perry (2004) | A Randomized School Trial of Environmental Strategies to Encourage Fruit and Vegetable Consumption among Children | Wrong concept |
| Hahnraths (2022) | The Effects of a Multi-Component School-Based Nutrition Education Intervention on Children’s Determinants of Fruit and Vegetable Intake | Wrong context |
| Hartson (2021) | Testing the Effects of Two Field-to-Fork Programs on the Nutritional Outcomes of Elementary School Students From Diverse and Lower-Income Communities | Wrong context |
| Gold (2014) | Discussion Map and Cooking Classes: Testing the Effectiveness of Teaching Food Safety to Immigrants and Refugees | Wrong concept |
| Gatto (2015) | LA Sprouts Randomized Controlled Nutrition and Gardening Program Reduces Obesity and Metabolic Risk in Latino Youth | Wrong context |
| Davis (2011) | LA Sprouts: A Gardening, Nutrition, and Cooking Intervention for Latino Youth Improves Diet and Reduces Obesity | Wrong context |
| Nicklas (2009) | Outcomes of a High School Program to Increase Fruit and Vegetable Consumption: Gimme 5 - A Fresh Nutrition Concept for Students | Wrong concept |
| van der Horst (2019) | Art on a Plate: A Pilot Evaluation of an International Initiative Designed to Promote Consumption of Fruits and Vegetables by Children | Wrong context |
| Jones (2012) | Food sustainability education as a route to healthier eating: evaluation of a multi-component school programme in English primary schools | Wrong context |
| Wang (2010) | Exposure to a Comprehensive School Intervention Increases Vegetable Consumption | Data unavailable |
| Mihrshahi (2017) | Evaluation of the Good Start Program: a healthy eating and physical activity intervention for Maori and Pacific Islander children living in Queensland, Australia | Wrong concept |
| Adab (2018) | The West Midlands ActiVe lifestyle and healthy Eating in School children (WAVES) study: a cluster randomised controlled trial testing the clinical effectiveness and cost-effectiveness of a multifaceted obesity prevention intervention programme targeted at children aged 6-7 years. | Data unavailable |
| Álvarez (2017) | Soporte nutricional y nutrición parenteral en el paciente oncológico: informe de consenso de un grupo de expertos | Wrong population |
| Pathirana (2017) | Impact evaluation of “Have Fun — Be Healthy” program: A community based health promotion intervention to prevent childhood obesity | Wrong population |
| Afsharnejad (2021) | KONTAKT® social skills group training for Australian adolescentes with autism spectrum disorder: a randomized controlled trial | Wrong context |
| Poll (2019) | Impact of intervention on nutritional status, consumption of processed foods, and quality of life of adolescents with excess weight | Wrong context |
| Schlange (2020) | Parent/Caregiver Perceptions of Youth Health Outcomes After Participating in the WeCook: Fun With Food and Fitness Program | Wrong context |
| Dialo (2014) | Participation des parents à un programme d’éducation nutritionnel implanté en milieu scolaire et développement de comportements alimentaires des enfants | Wrong population |
| Gans (1990) | Heart Healthy Cook-offs in Home Economics Classes: An Evaluation with Junior High School Students | Wrong concept |
| Asigbee (2020) | The Association Between Child Cooking Involvement in Food Preparation and Fruit and Vegetable Intake in a Hispanic Youth Population | Wrong concept |
| Garcia-Casal (2011) | A Program of Nutritional Education in Schools Reduced the Prevalence of Iron Deficiency in Students | Wrong context |
| Nursing Standard (1998) | Healthy eating programme gets the thumbs up from pupils | Data unavailable |
| Leuven (2018) | School gardening increases knowledge of primary school children on edible plants and preference for vegetables | Wrong concept |
| Mancone (2024) | Enhancing nutritional knowledge and self-regulation among adolescents: efficacy of a multifaceted food literacy intervention | Wrong concept |
| Ruge (2021) | student creativity and professional artwork in a school food intervention in Denmark | Wrong concept |
| Sharkey (2023) | Cooking with the Seasons for Health (CwS4H): An Innovative Intervention That Links Nutrition Education, Cooking Skills, and Locally Grown Produce to Increase Vegetable Intake among Limited-Resource Parent–Child Dyads in Rural Washington | Wrong context |
| Winham (2014) | Integration of Theatre Activities in Cooking Workshops Improves Healthy Eating Attitudes Among Ethnically Diverse Adolescents: A Pilot Study | Wrong concept |
| Bligh (2017) | A Sociocultural Analysis of Social Interaction and Collaboration Within the Cooking Practices of Children | Wrong concept |
| Lohse (2014) | resumo de conferência | Data unavailable |
| Dean (2022) | Cook Like A Boss: An effective co-created multidisciplinary approach to improving children’s cooking competence | Wrong context |
| Meng (2020) | resumo de conferência | Data unavailable |

Figure S1. Distribution of countries by studies on culinary interventions for children and adolescents in school settings (n = 55 studies)

Table S5. Recipes, techniques, and culinary skills developed in school-based cooking practice interventions for children and adolescents (n = 39 interventions)

| Intervention name (acronym) | Recipes, techniques, and culinary skills developed in the interventions. | |
| --- | --- | --- |
|  | Recipes | **Culinary techniques and skills developed** |
| Cookshops (CS)^37^ | Baked sweet potato with chili; broccoli and cauliflower (raw and steamed) with honey mustard dressing; greens sautéed with spinach, escarole, and kale; Hoppin' John; homemade salad dressing; pita; pumpkin sautéed with garlic and ginger; rye; three-bean and pepper vegetables; whole-grain bread (seeds); whole-grain rye bread | Food hygiene and safety practices; food waste reduction during preparation; knife handling |
| Health Education Intervention for Taste Development^38^ | Clear soup; miso soup; rice and bread; seaweed, mushrooms, and vegetables; soy, egg, fish, and meat | NR |
| Cooking With Kids^39,40,45,48^ | batatas persillade with cabbage; Chinese-American fried rice with vegetables; Indian lentils with carrot and raisin pilaf | NR |
| Stephanie Alexander Kitchen Garden Program (SAKG)^44,46^ | Homemade bread with herbs; broccoli sauce curry or homemade pasta with crystallized fruit; cake with orange and cardamom; green leaf salad; vegetable soup | Knife handling and food cooking techniques |
| Intervention program to encourage high school students to eat lunch at school ^42^ | chicken salad; tuna *wrap* | NR |
| Chefs Adopt a School Scheme (CAAS)^43^ | Pasta salad with tomato, cucumber, celery, peas, and red bell pepper | Food hygiene practices; food preparation practices; healthy eating information; sensory experience |
| Multifactorial Intervention for Hispanic Children at High Risk of Obesity^47^ | NR | knife handling and label reading. |
| Cooking and Active Leisure Tu y Alícia por la Salud (CAL-TAS)^49^ | Salad | NR |
| Food, Math, and Science Teaching Enhancement Resource (FoodMASTER)^50^ | Hot dog; hamburger; egg meringue; salmon pâté; cheese; vegetable soup | Cost of food; diet information; food hygiene and safety practices; food nature information; sensory and nutritional evaluation; food measurement techniques; recipe adaptation calculations and measurements; physicochemical reactions; teamwork. |
| Los Angeles Sprouts (LA Sprouts)^11,54,89^ | Apple with peanut butter; bean sauce with pita chips; breakfast taco; cucumber water; fresh vegetables with yogurt dressing; rainbow fruit with yogurt; seasonal green salad; supreme sandwich; *quesadillas* de legumes with pico de gallo; whole-grain pasta with vegetables; yogurt *parfait*; beet, carrot, and avocado salad; Jamaica water. | Assessment and reading: sugar evaluation; label reading; food origin; plant structure; Behavior: commensality; market shopping; Knowledge about food: seasonal foods; fiber; fruits; vegetable intake; colorful vegetables; Hygiene and safety: food hygiene; Preparation techniques: types of cooking. |
| Cooking Program For Alternative Education Students^51^ | Biscuit and homemade lemonade; breakfast burritos; chocolate mousse; curry with vegetables and chicken; spaghetti à la bolognese; vegetarian chili; walnut bread with fruit salad. | NR |
| Kids in the Kitchen^52^ | NR | Food identification; information on macro- and micronutrients; knife handling. |
| Jamie Oliver's Kitchen Garden Project (JOKGP)^53,58^ | Apple and blackberry crumble; baked stuffed bell peppers; cabotiá pumpkin muffins; focaccia with garlic and rosemary; homemade fish sticks; homemade mini hamburgers; tomato salad with tuna. | Knife handling; recipe adjustment; safety awareness. |
| Common Threads^55^ | NR | Food hygiene and safety; interpretation of recipes; knife handling; mise en place and cooking techniques; use and knowledge of spices and seasonings. |
| The Spice MyPlate^56^ | Chili hot dish; cinnamon yogurt *parfait*; electric pot spicy chicken; fruit fondue; homemade marinade sauce (nutmeg); homemade salad dressings; oatmeal with raisins and cinnamon; pepper sauce (rosemary*)*; *pita* stuffed with chicken; savory vegetable sauce; spicy chicken strips (cumin*)*; spicy trail mix; sweet trail mix; *My Plate* sauce; scrambled eggs with spices; spicy chicken; turkey *sloppy joes* (garlic). | Choosing healthier foods on a limited budget; cooking methods proficiency; flavor enhancement introduction; knife handling; kitchen safety; skills in using spices. |
| Taste Lessons (TL)^57^ | NR | Food hygiene practices; food guide reading; knife handling. |
| Teen Cuisine^59^ | Healthy chicken, nuggets, and French fries; eggs, scrambled and boiled; whole grain breads; yogurt and orange sauces. | Comparison of foods; creating daily meal plans; grain identification; knife handling; label reading; preparation of a simple, low-cost, and nutritious meal and/or snack; food safety; simple culinary terms and techniques; cooking and preparation techniques. |
| Cook Healthy Edible Food (CHEF)^60^ | NR | Basic food and kitchen safety rules; knife handling; snack preparation processes. |
| Cooking Bus^61^ | NR | Food hygiene; knife handling; technical, social, and linguistic aspects of food. |
| Choose Health: Food, Fun, and Fitness (CHFFF)^62^ | The recipes include at least one fruit or vegetable, and many feature whole grains and/or foods new to the participants. | Classifying nutrition labels by sugar, fat, and fiber using real food labels; measuring fat in fast foods; measuring sugar in sugary drinks. |
| iGrow (iGrow)^63^ | Healthy snacks; preparations with plant-based proteins; salad; leftover fresh vegetables. | Basic knife handling safety introduction; food safety. |
| Waves^64^ | NR | Knife and utensil handling for chopping, grating, peeling, and mixing foods. |
| Veggiecation^65^ | Bell pepper rice cake; cucumber salad; sweet potato salad; *daikon* kimchi cubes; mushroom *namul*; spinach *namul*; zucchini *namul*; tomato juice. | Knife and utensil handling for cutting, peeling, and mashing. |
| Nutritional intervention for children with mental and neurodevelopmental disorders ^66^ | *Bolonese* pasta; *Carbonara* pasta; fresh vegetable noodles; *Niçoise* salad; *Saltimbocca*; sautéed chicken; *Teriyaki* beef.. | Cooking techniques; food hygiene and safety; knife handling; use of thermometer. |
| Texas Sprouts (TX Sprouts)^9,67,72,74,79,81,85,86^ | Black beans; cucumber/radish hummus bites; cucumber mint water; whole grain pasta with vegetables; fruit rainbow; herb tea with spices and cinnamon; *legume* quesadillas with salsa; corn salad; lemon toast; fresh lemon water; pepitas. | Cooking safety; fiber and vegetables; knowledge of food systems (processed and unprocessed). |
| Exercício Gyotaku^68^ | Fried fish fillets | Fish cooking; fish filleting; tasting. |
| Culinary Education Program for Schoolchildren^69^ | Apple and turkey sandwich; chocolate cereals; croquettes with tuna; falafel; fruit salad; fruit yogurt; rainbow wrap; pancake with pineapple; pizza with vegetables; *pumpkin* whoopie; *muesli* Swiss-style; roasted zucchini and cauliflower sticks; salad with dressings; rice salad; smoothie; strawberry-frosted cupcake; fish nuggets; chicken nuggets; flatbread. | Beating, boiling, mixing, and baking; dough kneading/mashing; hand washing; knife and peeler handling; shaping; seasoning; types of cuts; use of grater; breading. |
| Garden-Based Integrated Intervention^70^ | Beetroot *tortillas*; bacon and scallion roll; carrot *canapé*; cheese and potato pancake; red radish sandwich; salad with vegetables; tofu wrap with lettuce. | NR |
| Practice-focused nutrition intervention in Hungarian^71^ | Apple muffins (traditional); apple muffins with whole wheat flour; banana and apple muffins; carrot yogurt cream; homemade fruit yogurt with banana; pumpkin soup; pumpkin soup with baked apple; pumpkin soup with baked apple and orange; creamy eggs; creamy ham; ham and cheese quesadilla; *panna cotta*; strawberry and banana muffins; spicy green cream cheese; tuna *tortilla* roll; traditional Hungarian sweet made with curd cheese. | Cooking; cup measurement use; knife handling; label reading; peeling; reading recipes. |
| EgizuSUK! Project^73^ | Broccoli and *empanadilla* pie; steamed broccoli with garlic sauté; spinach pie; spinach spring rolls; spinach salad. | Cooking techniques; knife handling. |
| School cooking program in Japan^75^ | Scrambled eggs with cabbage and cherry tomatoes; cabbage and tuna salad with cherry tomatoes; soup with cabbage, cherry tomatoes, and sausage. | Knife handling |
| KidChen^76^ | Fried brown rice; peanut butter and egg sandwich; rainbow of fruits; rainbow of vegetables. | Food hygiene; knife handling; kitchen safety; use of electric pots and equipment. |
| Professional Cooking (PC)^77^ | NR | Cooking and experiential learning: food safety and hygiene; measuring ingredients; preparing and cooking foods using different techniques; reading and following recipes. |
| Food Camp^78^ | Chicken and fish preparations; fruit yogurt; porridge with topping; vegetable bouquets. | Food guide reading; food preparation; technical visit. |
| Cooking Kit for Kids (COOKKIT)^80^ | Tuna pasta; vegetarian party; *Cuscuz Jóia*; *Rainbow Rumble*. | Cooking safety; fiber-rich ingredients and the benefits of low-calorie foods; knife and kitchen utensil handling; healthy food information; techniques for flavoring without salt. |
| Fuel for Fun (FFF)^82,83^ | American-Chinese fried rice with vegetables; Indian lentils with carrot and raisin pilaf; persillade potatoes with cabbage. | NR |
| Apprenti en Action^84^ | Apple purée; boiled eggs; *French toast*; granola; smoothie bowls. | Cooking techniques; knife and peeler handling; measuring cup use; reading recipes. |
| Pulses Make Perfect Sense (PMPS)^87^ | Cooked legume sauce and pasta/soup mix; cooked lentils; cooked soup mix; hybrid meat and legumes; lentil smoothies. | Cooking safety; cost information of foods; economical dish cooking; food storage; knife handling; macro- and micronutrient information. |
| Coordinated Approach to Child’s Health (CATCH)^88^ | Ants on a log; Apple and beetroot salad; broccoli smoothie; chickpeas; fruit pizzas; salad with three different leaves and dressing; stems and stalks. | NR |

Legend: NR – not reported.
